# Enhancing Thermal Efficiency in Water Storage Tanks Using Pigmented Recycled Concrete

**DOI:** 10.3390/ma17051008

**Published:** 2024-02-22

**Authors:** Jorge López-Rebollo, Ignacio Martín Nieto, Cristina Sáez Blázquez, Susana Del Pozo, Diego González-Aguilera

**Affiliations:** Department of Cartographic and Land Engineering, Higher Polytechnic School of Ávila, University of Salamanca, Hornos Caleros, 50, 05003 Ávila, Spain

**Keywords:** pigmented concrete, recycled concrete, thermographic properties, heat storage, water tank

## Abstract

The present work investigated the manufacture of elements such as water tanks from recycled concrete for applications where industries require water heating. This proposal leverages precast rejects for recycled concrete and incorporates colouring pigments. It is expected to contribute to the circularity of construction materials (due to the total replacement of natural aggregates by recycled aggregates) as well as to energy and emissions savings, which are attributed to improved thermal performance driven by the thermal behaviour that the coloration pigment gives to the manufactured concrete elements. To assess the efficacy of the proposed solution, on the one hand, mechanical tests were carried out in tensile, compression and modulus of elasticity, which showed a suitable concrete dosage for HA-30 structural concrete. Simultaneously, in search for a material that would increase the internal temperature of the tanks, thermal tests were carried out in a controlled laboratory environment on samples with different percentages of pigment, and an optimum concentration of 1% was obtained. It was also found that the thermal conductivity remained almost unaffected. Finally, two water tank prototypes were manufactured and tested under real environmental conditions: one with the optimised pigment concentration solution and other (the reference tank) without pigment. The results revealed that the colourised tank with the optimal concentration resulted in an average water temperature increase of 2 °C with respect to the reference tank. Finally, the economic and environmental benefits of this temperature increase were studied for industrial processes requiring water heating with a potential saving of 8625 kWh per month.

## 1. Introduction

With the aim of responding to the devastating effects of climate change, efforts have been focused towards mitigating greenhouse gas (GHG) emissions in all industrial and residential areas characterised by substantial fossil fuel and material consumption. In this context, considering the growing integration of renewable energy sources, the circular economy (CE) paradigm plays an imperative role. The core of CE is the “restorative resource utilization” concept, which seeks to eliminate the wastage of raw materials [1]. This approach not only provides new pathways for resources but also contributes to the effective management of the waste generated in initial activities.

The construction industry stands out as one of the most resource-intensive sectors, accounting for the significant generation of solid waste worldwide [2]. Construction and demolition waste (CDW) comprises different materials, such as bricks, concrete, wood, mortar, tiles or materials left unused during the construction process for external reasons. The recycling and reuse of such waste is favourable for its potential to mitigate pollution and decrease the demand of new natural aggregates, which in turn reduces energy consumption and CO_2_ emissions [3,4]. In this sense, the use of recycled concrete has garnered substantial attention across different applications. Given the nature of these compounds, it is essential to know their mechanical properties and limitations in order to determine their suitability in line with specific sample composition [5,6,7]. Among the different types of recycled aggregates, prior studies [8,9] have demonstrated the viability of precast concrete in the manufacture of structural elements, providing a sustainable alternative with an adequate performance.

In the quest for environmentally sustainable solutions, the incorporation of other additives into recycled concretes is a common practice. This aims to enhance different mechanical, structural or thermal properties of the base mixture while preserving its fundamental attributes. Depending on the intended application, several elements have been introduced into the concrete mix: paint-based coatings to reduce the temperature of facades and roofs [10]; waste or recycled materials to improve thermal conductivity and increase the thermal performance of buildings [11]; composites or phase change materials to increase the conductivity and improve the compressive strength for building applications [12]; mineral aggregates to improve the self-compaction of concrete at high temperatures [13]; and steel fibres, polypropylene or basalt to improve fracture properties [14,15].

Focusing on improving the thermal performance of concrete compounds, which aligns with the current emphasis on energy efficiency, these additives are designed to increase the energy efficiency of the spaces or structures where the concrete mixture is included. In this context, pigmented mortars emerge as a compelling solution due to their capacity to not only improve thermal characteristics (extending beyond the aesthetic appeal) but also ensure the preservation of the concrete’s mechanical properties [16]. The incorporation of pigments into concrete offers advantages compared to alternative options such as paints, owing to their greater durability and environmental attributes. Nevertheless, optimising the pigment dosage is imperative to minimise costs while maximising material performance [17].

Starting from a proper composition of the concrete and the inclusion of pigmented additives, the enhanced properties of the resultant compound can be of great contribution to the optimisation of energy consumption across different applications and sectors [18,19]. Depending on the intended use, these pigments can be strategically employed to either increase or decrease thermal absorption from external sources, which will have a direct impact on the internal environment. Therefore, this approach can lead to reduced cooling or heating requirements for a specific space or element, resulting in substantial cost savings and the derived environmental benefits.

This type of pigmented additive has traditionally been used for aesthetic or colorimetric purposes, such as in the case of heritage restoration [20]. Nevertheless, more recent works have investigated their use to improve some physical or mechanical properties of concrete: de Oliveira et al. pointed out the improvement of compressive strength by adding iron oxide-based inorganic pigments in concentrations of 2–5% [21]; Hatami et. al. improved the properties of coloured self-compacting mortars using intensely coloured nanoparticles [22]; Lermen et al. studied the substitution of sands with pigments from acid mine drainage as a sustainable alternative in civil construction [23]. Nevertheless, despite their potential in thermal applications, their use is very limited, so a comprehensive exploration of their influence on the thermal behaviour is imperative. To address this, the present research proposes a multidisciplinary investigation encompassing thermal conductivity measurements [24] combined with thermographic analysis [19]. In particular, the active thermography technique, widely used for evaluating a broad spectrum of materials [25,26], is here proposed. To simulate real-world conditions, a solar simulator was used to reproduce the environmental stresses conditions imposed by solar radiation [27].

Based on all the previously stated reasons, this paper aims to develop an innovative solution that enhances the energy and environmental performance attributes of concrete by combining recycled concrete and certain pigments. Specifically, this research pursues the optimisation of concrete pigment dosages to increase the internal temperature of the tanks by absorbing solar radiation. On the one hand, the total replacement of natural aggregates with recycled aggregates represents an economic and environmental saving in terms of materials and contributes to the circular economy. Furthermore, the heating of water due to the improved material properties saves energy and emissions in industrial applications that require water heating. In pursuit of this objective, concrete samples with different pigment dosages are manufactured in laboratory conditions. These samples are subjected to rigorous examination of their thermal behaviour during controlled heating facilitated by a low-cost solar simulator purpose-built with this aim. After identifying the optimal percentage of pigment for this purpose, a prototype water tank is built with this pigment dosage, and the thermal dynamics of the enclosed water is monitored under different external environmental conditions.

The manuscript is organised into the following sections. Section 2 describes the methodology and materials used for the testing and characterisation of the optimised pigmented concrete solution. Section 3 addresses the experimental laboratory tests performed on the pigmented concrete specimens as well as on the final water tanks. In Section 4, the optimised solution is revealed considering both economic and environmental aspects. And, finally, Section 5 briefly summarises the main findings of the research and outlines the expected future work.

## 2. Materials and Methods

**Figure 1:**
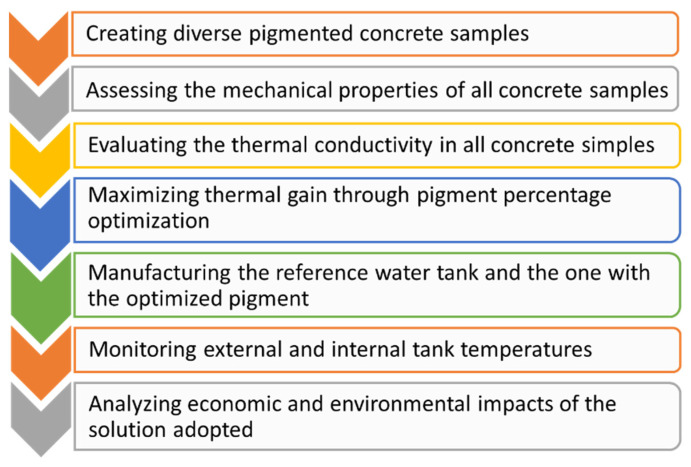
Workflow of the methodology and analysis performed.

To fulfil the objectives of this study, a systematic and rigorous methodology (Figure 1) was developed. This methodology has been designed to safeguard the integrity of concrete samples manufactured with varying pigment dosages, assuring their mechanical and structural stability. Additionally, it guarantees precision in the subsequent analyses, encompassing the thermal evaluation as well as the assessment of economic and environmental aspects associated with the water tank construction employing the optimal pigment dosage.

### 2.1. Pigmented Concrete Samples

This first phase focuses on the incorporation of pigments into the concrete manufacturing process with the goal of enhancing its thermal properties. Given the intended application of the proposed concrete tanks, the use of structural concrete, typically designed to withstand compressive stresses exceeding 25 MPa after 28 days of curing, is required [28]. Such concrete is generally composed of natural siliceous aggregates (both coarse and fine), Portland cement, and water. Nevertheless, the present research introduces a novel approach by substituting natural aggregates with recycled aggregates to promote environmental sustainability. These recycled aggregates come from rejected precast concrete elements, including concrete blocks, kerbs, and pipes that do not meet the minimum quality standards for market placement, which is usually due to production accidents or other factors. In this case, the recycled aggregates come from the precast concrete plant in Toro (Zamora, Spain), and they have been crushed and filtered through a 40 mm sieve in order to ensure proper sizing. Notably, the study employs a unified approach, replacing all the natural aggregates (both coarse and fine) with recycled aggregates.

Taking into account the origin of the aggregates, an exhaustive characterisation of the aggregates was not necessary. As they had already been used in the production of concrete, it was assumed that their shape, resistance to fragmentation, cleanliness and chemical composition met the quality standards. A particle size analysis was carried out to compare the particle size with the Fuller method. The particle size of the recycled aggregates corresponded to the ideal sizes, except between 2 and 1 mm, where there is a slight deviation between 6 and 14%. Nevertheless, these sizes have the least impact on the compactness and strength of the concrete, which allows these aggregates to be considered in the production of structural concrete.

The binder used in this case was a white cement of type BL I/B-LL 42.5 R [29], which is known for achieving a compressive strength of more than 20 MPa after two days and a compressive strength ranging from 42.5 to 62.5 MPa after 28 days. To provide a greater contrast with the pigmented mixtures, white cement with a whiteness content exceeding 85%, due to a very low content of metal oxides, was chosen.

Regarding the pigment, a black pigment HobbyColor by Europigments (Barcelona, Spain) was used. Taking into account that the purpose of the pigmentation is the highest heat accumulation, the black colour was chosen for a higher absorption of solar radiation. This synthetic pigment, composed of metal oxides, is inorganic and insoluble in water. The pigment has a composition of over 90% Fe_2_O_3_ with an average density of 4.60 g/cm^3^ and a predominant particle size of 0.15 microns. Unlike other coatings such as paints, this type of pigment exhibits resistance to alkalis and remains unaltered when exposed to sunlight or other atmospheric agents [30].

Consistency in concrete dosage was ensured throughout the study, encompassing mechanical tests, pigmented concrete formulations, and final manufactured tanks. Specifically, for every 100 kg of all-in-one aggregates, 30 kg of cement, and 16 L of water were added, yielding a water–cement ratio of 0.53. The water used for all the concrete manufacturing was non-aggressive water with a pH > 5, which is in accordance with the durability requirements of the standard used [28] and measured according to the Spanish UNE 83952 [31].

### 2.2. Mechanical Properties Characterisation

With the aim of analysing the suitability of concrete incorporating recycled aggregates for the construction of the test water tanks, a mechanical characterisation was carried out. Specifically, test were carried out to obtain compressive strength in accordance with the Spanish UNE-EN 12390-3 guideline [32], indirect tensile strength in accordance with UNE-EN 12390-6 [33], and modulus of elasticity in accordance with UNE-EN 12390-13 [34]. A Servosis electromechanical testing machine equipped with a 1500 kN load cell and corresponding compression platens was used for these tests.

Cylindrical specimens, with a diameter of 150 mm and a height of 300 mm, were manufactured in accordance with the Spanish UNE-EN 12390-2 guideline [35]. The specimens were kept in their moulds for 24 h under controlled conditions of 20 ± 5 °C. Subsequently, moulds were removed, and the specimens were transferred to a humid chamber maintained at a temperature of 20 ± 2 °C and a relative humidity exceeding 95% throughout the 28-day curing period.

It is noteworthy that for the mechanical tests, the concrete was manufactured without the inclusion of any pigment. Since the pigment percentage in all cases was equal to or lower than 10%, it was determined that the addition of pigment would not affect the concrete´s mechanical properties, as demonstrated in previous research [17].

### 2.3. Thermal Conductivity Test

Since the thermal performance of the material is to be analysed, it was decided to include the measurement of the thermal conductivity of the different samples prepared with different pigment concentrations. A significant variation of this parameter is not expected, but it is still considered interesting to know its behaviour, since it has been observed that this parameter varies considerably depending on the additive added, with significant changes at different dosages, even if the densities are kept similar [36,37].

In order to find out the thermal conductivity of the different manufactured mixtures, the transient line-source model [38] was selected as the theoretical substrate for the conducted test. This approach establishes an automated protocol to measure the heat dissipation provided by a linear source within a medium where thermal conductivity needs to be known. The temperature during the process was monitored, and the sample´s thermal conductivity was obtained using Equation (1):

(1) $$k=\frac{q}{m_{3}}$$

where *k* is the thermal conductivity, *q* signifies the heat flow and *m*_3_ is a constant derived in the process of measuring the evolution of temperature according to this model [39]:

Heating cycle (Equation (2)):

(2) $$T=m_{0}+m_{2}t+m_{3}\ln t$$

Cooling cycle (Equation (3)):

(3) $$T=m_{1}+m_{2}t+m_{3}\ln\frac{t}{t-t_{h}}$$

Here, *m*_0_ and *m*_1_ are the initial temperatures of the corresponding cycle, *m*_2_ is the rate of temperature variation, and *m*_3_ is the slope of the line resulting from plotting the variation in temperature against its logarithm.

**Figure 2:**
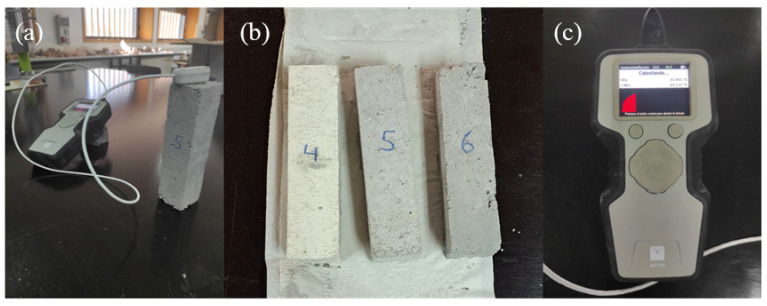
(**a**) Ongoing measuring process, (**b**) samples 4 to 6, (**c**) Decagon device recording the heating cycle.

For the thermal conductivity measurements, the TEMPUS model analyser from the company DECAGON DEVICES (Pullman, WA, USA) [40] (Figure 2), which complies with standardisation standards such as ISO 9000:2008 [41], ASTM D5334 [42], and IEEE 442 [43], was employed to ensure the rigour and reliability of the results. To connect the measurement and control device with the samples, the RK-3 sensor has been chosen. This sensor, measuring 60 mm in length and 3.9 mm in width at its widest point, requires the creation of corresponding drill holes in the samples for insertion. To ensure optimal thermal contact between the samples and the sensor, a mixture of diamond powder with thermal grease was used. The sensor operates within a conductivity range of 0.1 to 6 W/(m·K) and a precision of ±10%.

The measurements were conducted on 9 different samples, prepared as described in the correspondent Section 3.2, under constant ambient conditions at 22 °C. To minimise errors and significant deviations, the sensor was calibrated prior to each sample measurement, and a sufficient time interval between each of the 3 different measurements performed was ensured to achieve thermal equilibrium with the environment.

### 2.4. Maximising Thermal Gain through Pigment Percentage Optimisation

To optimise the appropriate pigment dosage, thermal tests were conducted, involving the exposure of different pigment concentrations to control heating using a solar simulator. The thermal response of these mixtures was analysed with a thermographic camera. The heating caused by the solar simulator and the absorption of solar radiation were monitored to compare the temperature reached by each of the samples with different percentages of pigment. Optimisation was carried out using the gradient descent method. This method is based on analysing the magnitude of the temperature increments to improve convergence to the optimal solution. In this case, the highest temperature increase was sought for the lowest pigment percentage increase.

**Figure 3:**
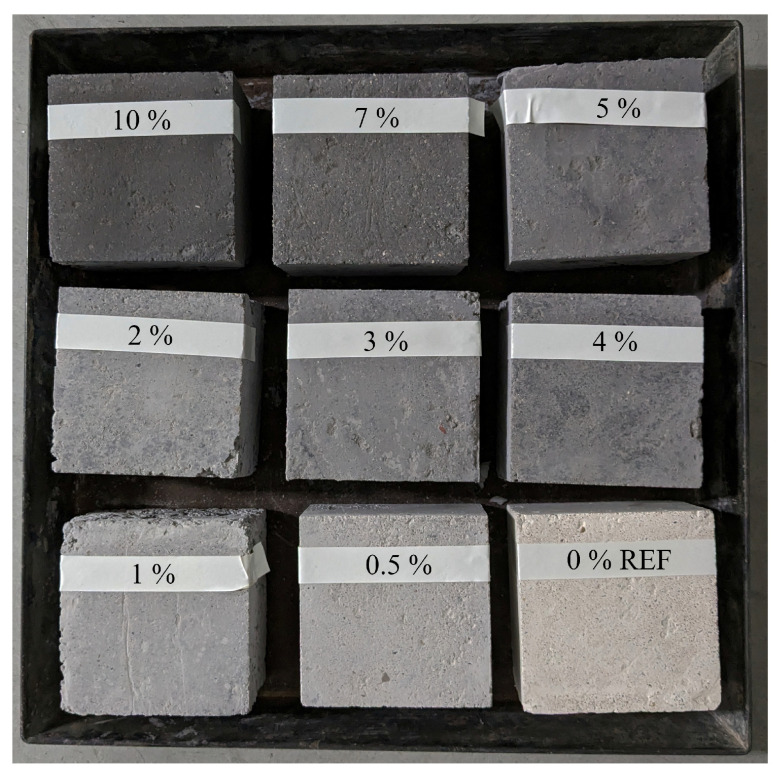
Samples for assessing the optimisation of pigment concentrations through thermal testing.

In total, 9 different pigment dosages were investigated taking into account the maximum amount recommended by the manufacturer (10%) (Figure 3): an initial reference sample devoid of pigment, and incremental dosages comprising 0.5%, 1%, 2%, 3%, 4%, 5%, 7% and 10% pigment relative to the cement content. Smaller increments were considered for lower percentages and larger increments were considered for higher percentages, as the greatest colour change occurs at the beginning until saturation. The dimensions of the samples were 100 × 100 × 50 mm to maximise the heated surface area, ensuring uniform and consistent testing conditions.

Monitoring the thermal behaviour of the 9 specimens involved the utilisation of specific equipment. Central to this process was an FLIR T540 (Wilsonville, OR, USA) thermographic camera (Figure 3), equipped with a 42° lens and an integrated RGB sensor, capable of capturing 30 frames per second. This camera offers a Thermal Infrared (TIR) resolution of 464 × 348 pixels and 5-megapixel visible (VIS) spectrum resolution. Additionally, it incorporates a laser sensor for pre-acquisition distance measurement, facilitating autofocus. Notably, the camera has a remarkable thermal sensitivity of less than 30 m·K at 30 °C, and it is calibrated for a temperature range from −20 to 120 °C. The FLIR software was used to manage the image acquisition, configure test parameters, control environmental conditions, and establish image capture protocols [11].

In the pursuit of replicating real-world environmental conditions for the concrete specimens under examination, a cost-effective solar simulator was custom-designed and employed. This setup allowed for the precise control of heat application to the specimens using an integrated lighting system. Consequently, this setup allowed to systematically monitor their subsequent heating and cooling phases by combining the thermographic camera with the solar simulator.

**Figure 4:**
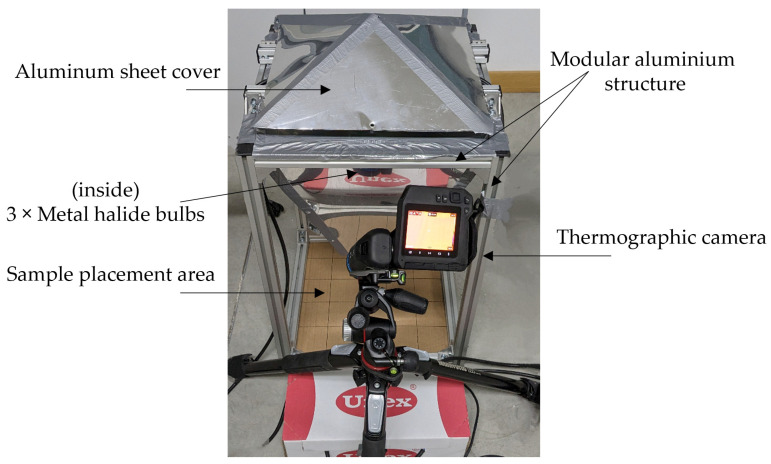
Temperature monitoring system consisting of a solar simulator and a thermographic camera.

The light source of the solar simulator (Figure 4) comprises three metal halide bulbs embedded in a prismatic aluminium structure with dimensions of 400 × 400 mm and a height of 700 mm. The design concentrates the lighting on the specimen. Specifically, three Philips Master HPI-T Plus 400W/645 E40 1SL/12 bulbs by Philips (Amsterdam, The Netherlands) [44] were used in conjunction with a ballast for precise regulation of arc current and voltage. This configuration was set after conducting a thorough lighting characterisation test [45], conclusively demonstrating that the stability and performance of this solar simulator were significantly enhanced when three bulbs were employed, as opposed to one or two. Additionally, the test confirmed the necessity of allowing a minimum of 5 min for light stabilisation to attain the maximum illuminance output from the cost-effective solar simulator. A spectrophotometer and a pyranometer were used for photometric and radiometric characterisation. According to the characterisation carried out, the output flux of the solar simulator was measured to be 270 W/m^2^ with an illuminance of 20,100 lx in the area where the samples were placed.

Prior to the initiation of the heating test, samples were kept isolated at a constant temperature of 22 °C, ensuring uniform initial conditions for each of the samples.

According to the specifications of the solar simulator, the device was powered up 10 min before placing the samples to achieve a stable illumination and spectral power distribution. Subsequently, samples were positioned at the centre of the incident area of the solar simulator, maintaining a vertical orientation at a distance of 50 cm. To minimise distortion, the thermographic camera was placed at the same distance with the least possible inclination.

The heating test started upon sample placement, and the thermographic image acquisition was initiated at a rate of 1 fps. The heating phase was maintained for 20 min, after which the solar simulator was turned off. Image acquisition was continued for an additional 40 min to capture the cooling phase, which was primarily driven by natural convection. It should be noted that despite efforts to maintain constant ambient temperature, heating by the solar simulator can induce slight alterations and oscillations during the cooling phase.

### 2.5. Manufacturing of the Water Tanks

Two prototypes of recycled concrete tanks were manufactured to carry out the experimental validation in real conditions. Although they were manufactured and subjected to real environmental conditions, a smaller scale than the industrial scale was used due to the limitations of the material and the study. Despite the complexity of direct scaling or extrapolation of results, the construction of two tanks of similar size will allow the comparative study that is the objective of this research.

**Figure 5:**
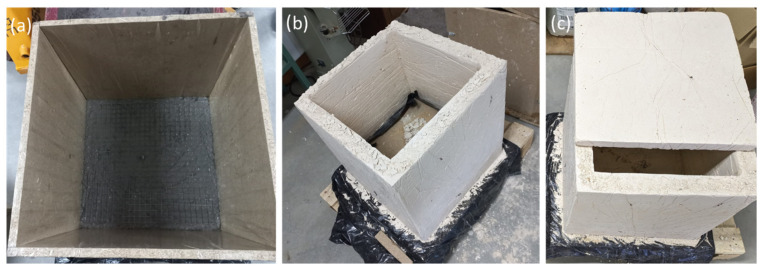
Manufacturing of tanks. (**a**) Formwork and rebar on the base, (**b**) finished tank in the absence of removing the base formwork piece, and (**c**) final reference tank with its lid.

The tank manufacturing process encompassed several critical stages conducted within a controlled laboratory setting. The initial phase was the design and warehouse planning, where attention was given to tank external dimensions (500 × 500 × 500 mm with a 50 mm thickness), shape, and the necessary rebar reinforcements. Notably, a steel grating reinforcement was devised for both the tank base and sides (Figure 5a).

Following the design phase, custom-cut wooden planks were employed to construct precision formwork moulds, integrating rebar reinforcements (Figure 5a). Subsequently, the concrete mixture was meticulously prepared using a concrete mixer, sequentially adding its components: water, cement, and pigment (only for the pigmented tank), which was followed by the introduction of recycled aggregates.

To ensure uniformity in the mixture, the concrete was carefully poured into the formwork, which was then subjected to vibration and compaction measures to mitigate air bubble formation. Subsequently, a well-established 7-day curing period was adhered to, ensuring that the concrete reached its peak strength. Finally, the wooden planks enclosing both the tanks and their lids were carefully removed (Figure 5b), unveiling the final tanks (Figure 5c).

**Figure 6:**
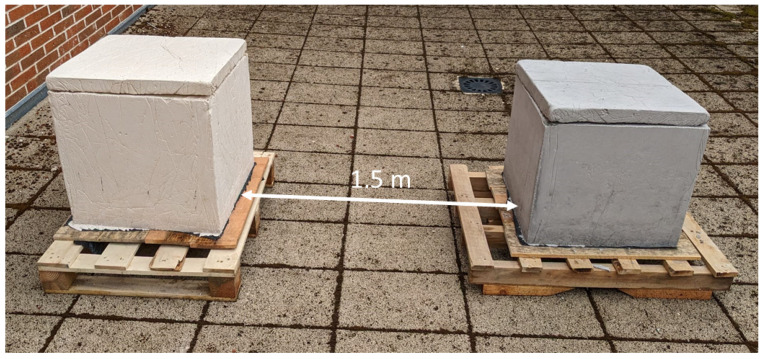
Geometric arrangement of the two tanks on the terrace.

To monitor the thermal behaviour of the water tanks and the influence of the pigmented dosage on the internal water temperature, both tanks were filled and placed in identical ambient conditions. Specifically, tanks were placed on an outdoor terrace at the roof of a building, maintaining a distance of 1.5 m to prevent casting of shadows (Figure 6). They were then filled with water, while water temperature thermometers were strategically positioned to monitor the internal water temperature for one month.

For this purpose, two DS18B20 by Analog Devices (Wilmington, MA, USA) submersible temperature sensors with a functional measuring range of −10 to 85 °C and decimal accuracy in absolute values were used. To minimise this error and ensure greater accuracy in measuring relative temperature increases, the instruments were calibrated to the same temperature prior to immersion. In addition, ambient temperature and humidity sensors were placed between the two tanks to monitor the surrounding environmental conditions. To ensure seamless data collection, all sensors were interconnected by sending real-time data at a 5 min interval through a LoRaWAN Gateway. In addition, a Grafana 10.0.0 platform was configured to visualise and manage the acquired data.

In addition to the water temperature monitoring, the surface temperature of the tanks was also monitored. For this purpose, thermographic images were taken with the thermographic camera described in Section 2.4. Given the time of the year in which the tests were performed and the slight inclination of the sun’s rays, it was established that the upper surface of the tank received the most sunlight exposure. Thus, vertical images were taken from a height of 1 m for each of the tanks. This capture process spanned 12 h with an image recorded every 40 min.

## 3. Experimental Results

### 3.1. Mechanical Properties

A total of 20 samples were subjected to mechanical tests. Ten of them were used to determine their compressive strength; following the guideline outlined in UNE-EN 12390-3 [32], the specimens were centred in the plates, and the test speed was set at 0.6 ± 0.2 MPa/s. Five of them were used to determine the indirect tensile strength; following the guideline UNE-EN 12390-6 [33], the specimens were placed in a horizontal position, applying a loading rate of 0.6 ± 0.2 MPa/s in a thin strip along their entire length. The other five samples were used to determine the secant modulus of elasticity in compression; following the guideline UNE-EN 12390-13 [34], three loading cycles were applied at a rate of 0.6 ± 0.2 MPa/s, ranging from 10% to one third of the compressive strength.

Once the mechanical properties was obtained for each specimen, essential statistical parameters, including the mean value, standard deviation, coefficient of variation to measure dispersion expressed as a percentage, and maximum and minimum values, were calculated. These results are presented in Table 1.

The tests yielded satisfactory results to consider the manufactured concrete as a structural concrete, since all the values were above 25 MPa. Furthermore, considering that the concrete was made of recycled aggregates, a coefficient of variation of about 10% was observed for the compressive and indirect tensile strength and around 16.5% was observed for the modulus of elasticity. This value can be considered acceptable for this particular type of concrete given the greater inherent heterogeneity of recycled aggregates when compared to natural aggregates [7]. Taking into account these results, it can be stated that the concrete can be used for structural purposes, which is classified as HA-30.

### 3.2. Thermal Conductivity Test

Three separate measurements of thermal conductivity were conducted for each sample. The results obtained for each of them, along with the mean values and the standard deviations, are presented in Table 2.

Upon careful examination of the results, it becomes evident that with the possible exception of sample 2, there was minimal variation in thermal conductivities among the samples, encompassing a range of approximately 0.25 W/(m·K). In general, this level of variance is within the acceptable range the measuring device employed.

To investigate these results further, an ANOVA test was performed to assess whether the incorporation of the pigment changed the thermal conductivity. A *p*-value of 0.00016 was obtained, indicating the existence of significant differences in the overall data. A one-way Tukey test was then performed to compare the results of the reference sample with the other pigmented samples, and the *p*-values obtained are shown in Table 2. These results show that the mean of the reference sample is not significantly different from the other samples (*p*-values > 0.05). The evidence provided by the ANOVA test refers to the fact that the mean of the sample with 0.5% pigment shows a significant difference as it is the sample with the lowest value and its *p*-value is less than 0.05. Considering that the recycled aggregate used to manufacture the concrete can introduce certain heterogeneity to exist in their local composition, we can conclude that the concentration of the additive has a negligible impact on the conductivity measurement.

These findings enable the analysis of the thermal behaviour under standardised conditions for all samples. In this way, any heating or cooling of the material will not be attributed to variations in thermal conductivity but will depend on the radiation absorption caused by the difference in pigment concentration.

### 3.3. Thermal Behaviour

The heating tests were conducted individually for each of the nine samples ensuring the same initial conditions as previously described.

Although the test considers both heating and cooling phases, the study focused mainly on the heating phase and the maximum temperature increase, as the aim was to find the sample that would enable the highest temperature accumulation.

For this analysis, thermographic images were analysed using a Region of Interest (ROI) covering the entire sample surface. This approach was justified by the uniform illumination provided by the solar simulator, rendering the consideration of edge effects during heating unnecessary [11].

**Figure 7:**
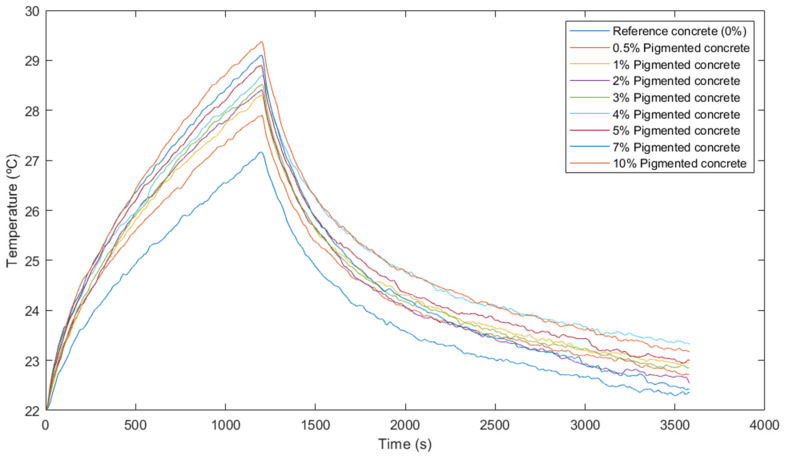
Heating and cooling curves of thermal tests.

A total of 3600 frames were obtained, 1200 for the 20-min heating phase and 2400 for the 40-min cooling phase. From each frame, the average surface temperature within the selected ROI was extracted to minimise the impact of potential surface irregularities leading to anomalous pixels. Then, the temperature evolution curves were generated, as shown in Figure 7.

Due to the high sensitivity of the camera and the effects introduced by the shutter and the rapid capture frequency, some frames presented variations compared to their neighbours. To overcome this, an attempt was made to smooth the curves by applying a moving average filter.

Once the temperature curves were plotted, the maximum temperature values reached during the heating phase were extracted for each sample, resulting in the temperature increase. Relative increases were then calculated for each sample, representing the temperature difference between a sample and the one with the lowest dosage. These findings are shown in Table 3.

In general, an increase in pigment dosage corresponded to a higher temperature rise at the end of the heating phase. The higher the pigment content, the darker the colour of the concrete. As dark materials have a greater capacity to absorb radiation in the visible and infrared wavelengths, the higher absorbed radiation in blacker concretes results in greater heating, as shown by spectral analysis in previous studies [17].

The results in Table 3 highlight the significant temperature increase observed for the first two dosages. The addition of 0.5% pigment led to a 14.3% increase in temperature rise compared to the non-pigmented dosage, while the 1% dosage improved this temperature increase by 6.9% compared to the previous dosage. Nevertheless, beyond the 1% dosage, the relative temperature increases diminished to less than 2%.

During the cooling phase, the temperature curves were practically similar for all dosages with no significant differences observed. Minor variations may be attributed to causes associated with the measurement process and the nature of cooling, potentially causing slight thermal oscillations caused by natural convection.

Considering the temperatures reached by each dosage and trying to achieve cost and resource efficiency, it was determined that the 1% dosage was the optimal solution to build the water tank.

### 3.4. Monitoring Water Tanks Temperatures

#### 3.4.1. Monitoring Surface Temperature

**Figure 8:**
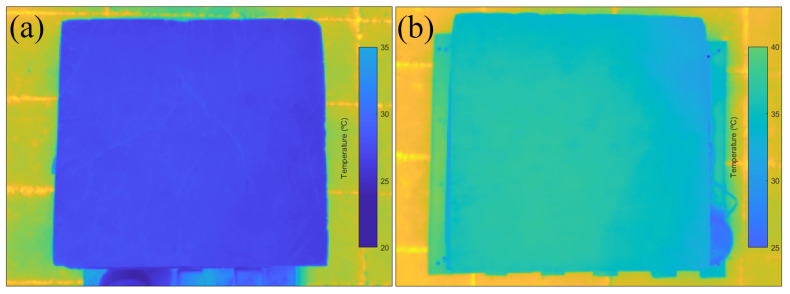
Thermographic images at the time of highest surface temperature for both tanks. (**a**) Reference tank and (**b**) pigmented tank.

A comprehensive analysis of surface temperature was conducted involving the capture of 19 pairs of images from the reference and from the 1% dosage pigmented tank (Figure 8). These image pairs were taken at 40-min intervals over a 12 h period, coinciding with sunrise and sunset.

For each image, the same procedure as that employed in the previous thermographic analysis was followed. This process involved extracting the average temperature from the ROI corresponding to the upper surface of the tank.

**Figure 9:**
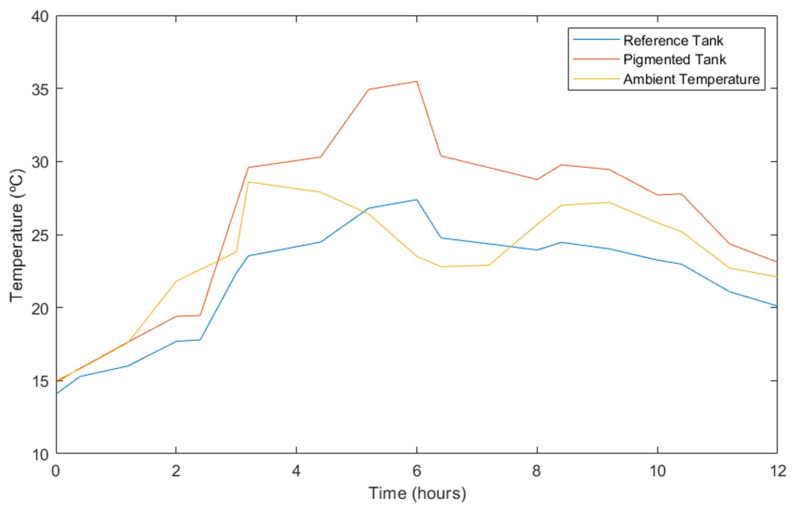
Evolution of tank surface and ambient temperature.

The temperature obtained from these thermographic images was correlated with the ambient temperature at the time of each corresponding capture; its evolution over time is shown graphically in Figure 9.

According to Figure 9, a significant disparity exists between the surface temperature of the reference tank and the one pigmented. While the pigmented tank reached a maximum temperature of 35.5 °C, the reference tank only reached 27.3 °C. In other words, there was an 8.2 °C difference in surface temperature. Furthermore, the reference tank did not exceed the maximum ambient temperature.

As mentioned in the previous section, the effect of the pigment is based on the increased absorption of solar radiation, which causes an increase in the temperature of the material, which is in this case measured at the surface. Meanwhile, in the laboratory samples, a temperature difference of 1.15 °C was reached after 20 min of heating between the reference concrete and the concrete with 1% pigment; in real conditions, this difference was seven times higher and was reached after about 6 h. Nevertheless, heating in this case was not homogeneous and constant, as more complex external factors could intervene during this process, such as ambient temperature, wind cooling and the fact that solar radiation is not constant due to the movement of the sun and the possible presence of cloud cover.

Additionally, upon comparing the surface temperature evolution with the corresponding ambient temperature, it was observed that both tanks reached their maximum surface temperatures approximately two hours after the ambient temperature peaks. Furthermore, in the case analysed, a temporal correlation between a decline in ambient temperature during the day and a corresponding decrease in surface temperature was observed. Afterwards, as the ambient temperature raised again, there was a slight increase in surface temperature.

For most of the observation period, the surface temperature of the pigmented tank was above the ambient temperature, while the reference tank consistently registered lower temperatures than the ambient environment. Nevertheless, it should be mentioned that the measurements did not account for cloud cover. The presence of cloud cover at certain times could potentially influence surface temperature fluctuations, as it may obstruct the sun’s rays from heating the tank surfaces.

#### 3.4.2. Monitoring Water Temperature

**Figure 10:**
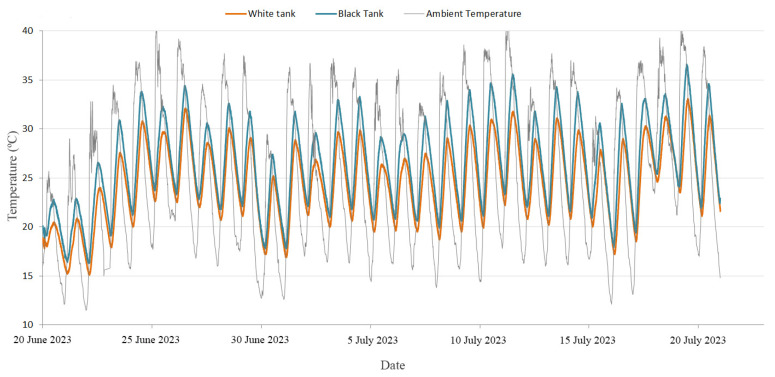
Registers of the water temperature in the developed tanks and the ambient temperature during the established 30-day period.

As part of the research, water temperature measurements were conducted within each of the tanks. For this purpose, the above-described sensors installed in the tanks were used to capture data at 5-min intervals. In this sense, Figure 10 shows a comprehensive depiction of the water temperature measurements taken over a 30-day period in both tanks. This figure also includes records of the ambient temperature, which were collected at the same time intervals.

**Figure 11:**
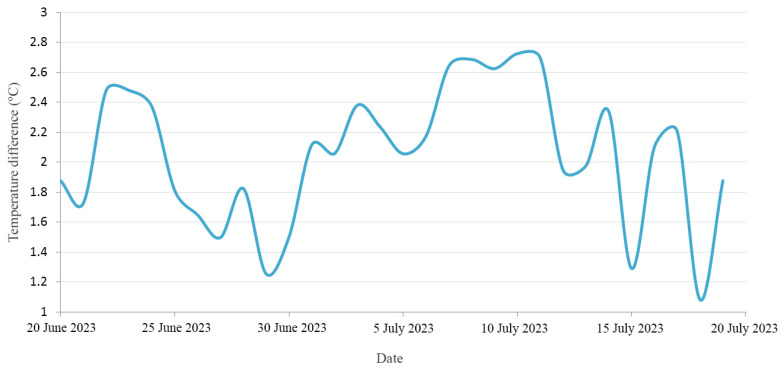
Evolution of the average daily differences in the water temperatures of both tanks.

In addition to the data presented in Figure 10, it is crucial to evaluate the temperature differences observed in the water accumulated in both tanks especially during periods of exposure to solar radiation. Taking into account the solar radiation hours in the study area, the daily average temperature difference during this solar period was calculated. Figure 11 illustrates the temporal evolution of these temperature differentials in water over the 30-day analysis period. Based on these values, the overall average water temperature in the tanks during the considered solar period was 1.98 °C.

## 4. Economic and Environmental Analysis

The results of this research suggests that under specific seasonal climatic conditions, a temperature increase of 1.98 °C can be obtained in the water stored by the tank with the optimal pigmented mixture. This can mean energy savings in thermal processes requiring heated water. For every litre of water, the energy savings needed for these thermal processes can be estimated at 8.05 J. Depending on the method chosen to achieve the temperature increase can lead to varying economic savings.

To quantitatively assess thermal performance and emission reduction, a segment of the agri-food industry was selected as a reference for water consumption in thermal processes. An estimate of approximately 45,000 cubic meters of water annually, evenly distributed over 12 months, can be attributed to a typical plant in the fruit-and-vegetable processing industry [46]. Considering that this work focused on a one-month period, achieving the proposed benefits would require an appropriate scheduling of processes. With a monthly water consumption of 3750 m^3^ for medium-temperature thermal processes, the energy savings, based on the initial water temperature difference, amount to 31,066.2 MJ (8625 kWh). The corresponding economic and environmental savings, including CO_2_ emissions, considering different energy sources, are shown in Table 4.

Due to the changing climatic conditions in central Spain throughout the year, the data in Table 4 cannot be extrapolated to annual yields and emissions savings. Nevertheless, it can be noted that the calculations use an average temperature difference between the two tanks, and with an optimised process schedule, even greater temperature differences can be achieved. Furthermore, the average thermal difference may vary in other months of the year. In this context, future research will encompass longer monitoring periods, spanning one or several years, to analyse temperature evolution in the tanks further.

Considering that the average energy required to produce a conventional concrete element of 1 m^3^ is 29.4 kWh [49] and that the energy saved in the reference month is 8625 kWh, the inclusion of an analysis of the energy used to produce the pigment does not seem very relevant. The largest energy consumption in recycled concrete is related to the production of cement, the pigment represents only 1% by weight of this component, and the energy consumption in its production is much lower. It should also be noted that the addition of pigment is a one-off energy cost as opposed to a cumulative saving over time (e.g., over a lifetime of about 25 years).

Regarding the CO_2_ emission savings related to water heating, it is essential to highlight the environmental benefits of using recycled aggregates in concrete production. Conventional coarse and fine aggregates are associated with emissions of approximately 54 kg per cubic meter of concrete, while recycled aggregates are linked to emissions of around 30 kg [50]. This signifies a reduction of 24 kg of CO_2_ per cubic meter of concrete along with a preservation of 1100 kg/m^3^ of natural resources and a reduction in waste destined for landfills.

## 5. Conclusions

This research focused on investigating the thermal properties of recycled concrete with the aim of optimising the percentage of pigment as an additive to enhance its thermal performance. Experimental laboratory tests were complemented by tests on prototype water tanks to facilitate the comparison between the optimised solution and a reference one. This research carries a twofold significance. On the one hand, it advances the cause of sustainable construction by promoting the use of recycled materials, thereby contributing to waste reduction in the construction industry. On the other hand, it enhances the competitiveness of these materials, offering economic and environmental savings for industries reliant one water heating processes.

To begin, mechanical characterisation tests determined that concrete composed of recycled aggregates from precast waste can achieve structural properties and can be used for the manufacture of the proposed tanks, as it can be categorised as HA-30 structural concrete. Nine different mixtures were produced using this recycled concrete and black pigment: one as a reference without pigment and the remaining eight incorporating black pigment in percentages ranging from 0.5 to 10%.

Thermal conductivity tests demonstrated that the percentage of pigment had negligible effects on this conductivity properties with minor variations attributed to the heterogeneity of the recycled aggregate composition.

Thermal tests were carried out in the laboratory by monitoring the heating and cooling of samples using a low-cost solar simulator. These tests allowed for the selection of the optimal mixture using the gradient descent method, which was found to be 1% pigment. Under these conditions, the optimal mixture reached a temperature 1.15 °C higher than the reference mixture due to the greater radiation absorbed by the pigmented effect. This solution verified the initial hypothesis of higher heating for the pigmented concrete and also provided the highest temperature increases in relation to the percentage of pigment used.

Subsequently, two prototype water tanks were fabricated, one with the optimised pigment dosage and the other with the reference mixture, and exposed to ambient conditions for one month. Surface temperature monitoring revealed temperature differences of up to 8.2 °C for the optimised solution. The water temperature indoors was also notably higher, especially during sunny hours, with an average increase of 1.98 °C. Although the higher radiation absorption resulted in a significant increase in the surface temperature of the material, this was transferred to the water to a lesser extent due to its high specific heat and the low thermal conductivity of the concrete as well as other external factors such as ambient temperature or wind cooling.

The study also involved an economic and environmental evaluation, considering costs for industries requiring water heating. The savings associated for the one-month study indicated potential savings of 8625 kWh, which can be translated into substantial economic savings depending on the energy typically used.

In conclusion, this research highlighted the advantages of using both waste materials in concrete manufacturing and pigment for the enhancing thermal performance. Future investigations will expand to year-long monitoring and in-depth studies, facilitating comparisons between seasons. These analyses will help demonstrate the practicality of this solution, particularly in cold regions during winter months, where it could prevent water freezing. Additionally, forthcoming research will explore modifications to thermal conductivity properties to facilitate the transfer of higher surface temperatures to the water and will also incorporate numerical simulations to further refine the prototype under real environmental conditions and on an industrial scale.

## Figures and Tables

**Table 1:** Results of the mechanical characterisation.

Property	Mean	STD	CoV (%)	Minimum	Maximum
f_c_	38.5	3.87	10.0	29.1	43.5
f_ti_	2.8	0.3	11.2	2.5	3.1
E	27,615.5	4562.0	16.5	21,179.9	36,816.0

f_c_ = Compressive strength (MPa). f_ti_ = Indirect tensile strength (MPa). E = Secant modulus of elasticity in compression (MPa).

**Table 2:** Thermal conductivity results.

Sample	Measure 1	Measure 2	Measure 3	Mean Value	Std. Dev.	*p*-Value
Ref	1.14	1.11	1.14	1.13	0.01	-
0.5%	0.81	0.91	0.76	0.83	0.08	0.0999029
1%	1.41	1.43	1.41	1.41	0.01	0.1364058
2%	1.26	1.08	1.09	1.14	0.10	1.0000000
3%	1.26	1.52	1.47	1.41	0.14	0.1364058
4%	1.38	1.28	1.28	1.31	0.05	0.6234869
5%	1.29	0.99	1.01	1.09	0.17	0.9999902
7%	1.58	1.21	1.23	1.34	0.21	0.4596789
10%	1.43	1.29	1.18	1.30	0.13	0.7054091

Units in W/(m·K).

**Table 3:** Temperature and temperature increase in concrete samples in thermal tests.

Nomenclature	Maximum Temperature	Maximum Temperature Increase	Relative Increase
Ref	27.16	5.16	-
0.5%	27.91	5.91	0.74
1%	28.31	6.31	0.41
2%	28.41	6.41	0.10
3%	28.52	6.52	0.10
4%	28.70	6.70	0.19
5%	28.90	6.90	0.20
7%	29.11	7.11	0.20
10%	29.38	7.38	0.27

Units in °C.

**Table 4:** Prices and monthly savings per energy sources [47].

Energy Source	Price (€/kWh)	Monthly Saving (€)	CO_2_ Emissions Saved (kg) *
Electricity	0.18	1552.5	2854.9
Natural gas	0.07	603.8	2173.5
Gasoil	0.09	776.3	2682.3
LPG	0.08	690.0	2190.6

* For the estimation of the emissions saved with electricity as energy source, a standard location has been selected to consult the existing electricity mix and to establish an emissions factor per kWh of energy consumed. The electricity mix used was the conventional Spanish peninsular mix [48].

## Data Availability

Data are contained within the article.
